# Contrasting Classical and Machine Learning Approaches in the Estimation of Value-Added Scores in Large-Scale Educational Data

**DOI:** 10.3389/fpsyg.2020.02190

**Published:** 2020-08-21

**Authors:** Jessica Levy, Dominic Mussack, Martin Brunner, Ulrich Keller, Pedro Cardoso-Leite, Antoine Fischbach

**Affiliations:** ^1^Luxembourg Centre for Educational Testing, University of Luxembourg, Esch-sur-Alzette, Luxembourg; ^2^Department of Behavioural and Cognitive Sciences, University of Luxembourg, Esch-sur-Alzette, Luxembourg; ^3^Department of Education, University of Potsdam, Potsdam, Germany

**Keywords:** value-added modeling, school effectiveness, machine learning, model comparison, longitudinal data

## Abstract

There is no consensus on which statistical model estimates school value-added (VA) most accurately. To date, the two most common statistical models used for the calculation of VA scores are two classical methods: linear regression and multilevel models. These models have the advantage of being relatively transparent and thus understandable for most researchers and practitioners. However, these statistical models are bound to certain assumptions (e.g., linearity) that might limit their prediction accuracy. Machine learning methods, which have yielded spectacular results in numerous fields, may be a valuable alternative to these classical models. Although big data is not new in general, it is relatively new in the realm of social sciences and education. New types of data require new data analytical approaches. Such techniques have already evolved in fields with a long tradition in crunching big data (e.g., gene technology). The objective of the present paper is to competently apply these “imported” techniques to education data, more precisely VA scores, and assess when and how they can extend or replace the classical psychometrics toolbox. The different models include linear and non-linear methods and extend classical models with the most commonly used machine learning methods (i.e., random forest, neural networks, support vector machines, and boosting). We used representative data of 3,026 students in 153 schools who took part in the standardized achievement tests of the Luxembourg School Monitoring Program in grades 1 and 3. Multilevel models outperformed classical linear and polynomial regressions, as well as different machine learning models. However, it could be observed that across all schools, school VA scores from different model types correlated highly. Yet, the percentage of disagreements as compared to multilevel models was not trivial and real-life implications for individual schools may still be dramatic depending on the model type used. Implications of these results and possible ethical concerns regarding the use of machine learning methods for decision-making in education are discussed.

## Introduction

### Value-Added Modeling

Value-added (VA) models are statistical models designed to estimate school (or teacher) effectiveness based on students’ achievement. More specifically, they intend to estimate the “value” specific schools (or teachers) add to students’ achievement, independently of students’ backgrounds (e.g., Amrein-Beardsley et al., 2013). VA scores are estimated by juxtaposing the actual achievement attained by students attending a certain school with the achievement that is expected for students with the same starting characteristics (e.g., pretest scores).

The use of VA models is highly consequential because VA scores are often used for accountability and high-stakes decisions to allocate financial or personal resources to schools (for an overview from a more economical point of view, see, Hanushek, 2019). These high stakes can make estimating VA scores a politically charged topic, especially in the US, where many states have implemented VA-based evaluation systems (Amrein-Beardsley and Holloway, 2017; Kurtz, 2018). In 2015, 41 states recommended the use of VA scores or other student growth measures for human resource decisions (Kurtz, 2018). However, in recent years, the consequential use of VA models seems to be decreasing again in many states (Close et al., 2020).

Despite both the practical and political relevance, there is currently no consensus on how to best estimate VA scores (Everson, 2017; Levy et al., 2019). This lack of consensus can be observed for various aspects of the VA model, including the applied statistical model, methodological adjustments (e.g., for measurement error or missing data), and the selection of covariates used to compute the VA score. In the present study, we focused on how the choice of the statistical model affects VA scores, which are used to evaluate the effectiveness of schools, as VA scores and thus measures of school effectiveness may vary greatly depending on the statistical method used (e.g., Sloat et al., 2018). In particular, we compare classical models for prediction with those drawn from machine learning.

### Statistical Models for the Estimation of School VA Scores

While VA models stem originally from economics (Hanushek, 1971), they consist of statistical methods that are common in educational or psychological sciences. Even though there are many different possible statistical models, all VA models follow the same logic. As shown in Eq. 1, this expected achievement $\hat{y}$ is estimated for every student *i* in school *j* as a function *f* (e.g., linear regression) of their initial characteristics *x*_*ij*_ at an earlier time point (e.g., prior achievement) and an error term *e*_*ij*_.

(1) $$\begin{array}{c} {\hat{y}}_{i\,j}=f\,\left(x_{i\,j}\right) \end{array}$$

In a second step and as demonstrated in Eq. 2, the VA score for each school *j* is estimated by calculating the mean difference (i.e., residuals) between the expected achievement $\hat{y}$ and the actual achievement *y* for all *n* students in this school *j.* This is equal to the average error term *e* of all students in school *j*.

(2) $$\begin{array}{c} V\,A_{j}=\frac{\sum_{i}^{j}\left(y_{i\,j}-{\hat{y}}_{i\,j}\right)}{n_{i\,j}}=\frac{\sum_{i}^{j}\left(e_{i\,j}\right)}{n_{i\,j}} \end{array}$$

Positive VA_j_ values mean that students in school *j* achieved better than expected, while negative VA_j_ values mean that they achieved worse than expected. The aim is to statistically eliminate all factors that cannot be influenced by a school, such that everything that is left (i.e., the residuals) will be attributed to the effect of a certain school. Hence, the quality of the initial prediction step (i.e., Eq. 1) is crucial for the estimate.

#### Classical Approaches in the Estimation of Value-Added Scores

There are currently two main classical models to compute VA scores: linear regression and multilevel models (Kurtz, 2018; Levy et al., 2019). These models are often claimed to be interpretable for most researchers and practitioners (see, e.g., Molnar, 2020). However, they make strong assumptions (e.g., linearity), which may limit their accuracy. Intuitively, most people would agree that learning does not happen linearly (e.g., as illustrated in this blog entry, McCrann, 2015). This is underlined by findings that at least in some cases, non-linear models fit the data better than linear models, implying that the typical linearity assumption might not be warranted (Lopez-Martin et al., 2014). However, this does not necessarily mean that non-linear models are also more appropriate for the estimation of VA scores. For example, one finding from a national project on VA modeling was that even though the data fit was better when using a curve rather than a straight line, this had almost no effect on VA scores (Fitz-Gibbon, 1997). In situations with high noise, low model complexity can have better performance (Friedman et al., 2001), but as data quality and amount improve more complex methods may be more appropriate.

#### Machine Learning Approaches for the Estimation of Value-Added Scores

In educational research, as in many other domains, the amount of available data is consistently growing (as reflected in the development of the new domain of “educational data mining”; see, e.g., Romero and Ventura, 2010; Baker, 2019). Although big data is not new in general, it is relatively new in the realm of social sciences and education, requiring new data analytical approaches. This is both a challenge and an opportunity, as it is becoming feasible to use the strength of interdisciplinary approaches and combine expertise from the domains of Education, Psychology, and Computational Sciences to apply machine learning methods to estimate VA scores. While machine learning seems to be promising for practices within the classroom (see, e.g., Kaw et al., 2019; Moltudal et al., 2020), the focus of the present study is on their potential use for the estimation of school VA scores.

The collaboration of educational, psychological, and data scientists offers an alternative approach to the classical models: machine learning methods. Machine learning (ML) has yielded spectacular results in numerous fields, such as automated face identification (Taigman et al., 2014) or beating human players at the game Go (Silver et al., 2017). The fields of statistics and machine learning highly overlap in terms of tools and methods, primarily differing on focus of problems and applications (for a discussion, see, e.g., Harrell, 2018). With larger data sets, the social sciences are now drawing more on machine learning approaches (e.g., as in computational social sciences, Lazer et al., 2009), which may provide higher prediction accuracies. Often these approaches draw from more general non-linear function fitting approaches (e.g., kernel methods) or combine several weaker models to improve performance (e.g., boosting or random forests). However, these approaches often require large datasets and involve models that can be more difficult to interpret (i.e., black boxes).

Recent research in learning analytics and educational data mining has applied, with great success, machine learning to a wide set of educational problems (Romero and Ventura, 2020), such as predicting student performance in higher education (for recent reviews of the literature, see, e.g., Hellas et al., 2018; Papadogiannis et al., 2020). While a wide variety of models have been tested (e.g., decision trees, neural networks, support vector machines, and linear regression; Rastrollo-Guerrero et al., 2020), there is no consensus yet on which model is the “best” (Papadogiannis et al., 2020). This state of affairs is partially due to studies using different covariates or model performance metrics (Hellas et al., 2018). Such differences between studies make direct comparison of their results difficult, and may lead to inconsistencies and confusion (Papadogiannis et al., 2020). The lack of standardized benchmarks means that while it is clear that these machine learning methods may overall perform well in predicting student performance, determining which specific model to privilege requires direct statistical comparison on a given dataset. In addition, one should note that most predictive models are influenced by their modelers (see, e.g., Kuhn and Johnson, 2013), which begs the question of how far the VA scores from these predictive models differ from those obtained via more classical approaches.

The specific fruitfulness of machine learning methods for the application of school VA models is supported by recent research reporting higher accuracy and more reliable estimates of school VA scores when comparing “random forests” regression to a classical linear regression (Schiltz et al., 2018). To the best of our knowledge, this is the only study that has compared machine learning methods to a classical approach for the estimation of school VA scores. In Schiltz et al. (2018), simulated data and population data from Italy were used to investigate the application of random forests for the estimation of school VA scores. They reported that random forest models predicted outcomes more accurately than linear regression models. Not only did VA scores differ numerically depending on the model type used, the ranking of VA scores across schools differed as well (in particular among schools that ranked very high or very low) which implies that the choice of model type may have substantial practical consequences. The authors recommended the use of random forests over linear regressions when estimating school VA scores, especially when using VA scores for high-stakes decisions, as higher accuracy may prevail over transparency. Random forests methods can capture complex non-linear relationships between dependent and independent variables and are far more flexible than linear regression models; if the data deviates from linearity and the dataset is large enough, techniques like “random forests” can grasp patterns that classical linear models cannot.

This means that these random forests have an advantage over the classical linear regression model, as they do not assume a linear relationship. However, random forests only represent one type of machine learning approach, and so it is unclear whether the improved performance is due to either the non-linearities or the ensemble nature of the method. Additionally, linear regressions are only one of the two typically used model types in the estimation of school VA scores (Kurtz, 2018; Levy et al., 2019); the other one, multilevel models, was not considered in their study. Finally, it is unclear how their result will generalize across other datasets, in particular given differences in covariates and populations.

Hence, we expand on this work by considering a broader class of predictive models, which will be described in detail in the method section. In brief, we compare: linear, multilevel, and polynomial regression, random forest, neural networks, support vector machines, and boosted approaches (see also Table 1 for an overview).

**TABLE 1:** Description of the applied models.

Model	Relationship	Specifics	Package and function	Hyper parameters
Classical approaches				
Linear regression	Linear		*stats* (R Core Team, 2019)*: lm*	/
Multilevel model	Linear	Hierarchical structure taken into account	*lme4* (Bates et al., 2015)*: lmer*	/
Polynomial regression	Non-linear	Third degree to all continuous variables	*stats* (R Core Team, 2019)*: lm*	/
Machine learning approaches				
Random forest	Non-linear	Extension of decision trees	*ranger* (Wright et al., 2020)*: ranger*	– Randomly selected predictors: 2, 5, 8 – Splitting rule of variance, extra trees, maxstat – Minimum node size: 5, 8, 10.
Neural networks	Non-linear	Sequential logistic regression	*nnet* (Venables and Ripley, 2002)*: nnet*	– Number of hidden units: 1, 3, 5, 10 – Weight decay: 0, 0.001, 0.1, 0.5, 0.9
Linear support vector machines	Linear	Extension of regression approaches; combination of finding the minimal margin hyperplane and the kernel method	*kernlab* (Karatzoglou et al., 2004)*: svmLinear*	– Cost of constraint violation: 0.001, 0.01, 0.1, 0.5, 0.9, 1
Polynomial support vector machines	Non-linear		*kernlab* (Karatzoglou et al., 2004)*: svmPoly*	– Polynomial degree: 1, 2, 3 – Distance measure for kernel: 0.001, 0.010, 0.100 – cost of constraint violation: 0.001, 0.01, 0.1, 1
Radial support vector machines	Non-linear		*kernlab* (Karatzoglou et al., 2004)*: svmRadial*	– Distance measure (kernel): 0.01, 0.05, 0.1, 0.5, 1 – Cost of constraint violation: 0.001, 0.01, 0.1, 0.5, 0.9, 1
Boosting	Linear	Ensemble method; models sequentially trained based on performance of past models	*xgboost* (Chen et al., 2019)*: xgbLinear*	– Number of boosting iterations: 25, 50, 100 – L1 and L2 regularization: 0, 0.01, 0.01, 0.1, 1 – Learning rate: 0.05, 0.1, 0.3, 0.6

### The Present Study

As mentioned above, there is currently no consensus on how to best estimate school VA scores (e.g., Levy et al., 2019, but see also Schiltz et al., 2018). One previous study has sought to analyze systematically different covariate combinations in school VA models (Levy et al., 2020), with one limitation of this study being the use of only one model type (i.e., multilevel model). The present study thus aims to expand the study by Levy et al. (2020) by examining different model types for the estimation of school VA scores by the interdisciplinary approach of adding methods typically used in computational sciences to the typically psychometric approaches.

We aim to extend the study from Levy et al. (2020) by examining different model types for the estimation of school VA scores, and the study from Schiltz et al. (2018) by using a different data set with population data, by adding multilevel models, by adding non-linear “classical” models, and by adding different types of machine learning methods (e.g., with and without the assumption of linearity) to the comparison.

A common and appropriate way of comparing predictive models is by using a class of methods called cross-validation (Hastie et al., 2009). Cross-validation allows us to estimate a model’s out-of-sample performance, that is performance on predicting data that the model was not fit on. It achieves this by randomly splitting the data into “train” and “test” subsets. The model is then fit on the training set, and performance (e.g., *R*^2^) is evaluated on the testing set. This process can then be repeated, either by randomly subsampling or by an initial partitioning, allowing the results to be averaged. For all models used in our analysis, VA scores were computed based on average residuals per school, in the same way as the linear model.

For our analysis, we used the same selection of covariates across all statistical models. This allows for a fair comparison between models. The choice of covariates was made based on the basis of models of school learning (e.g., Haertel et al., 1983; Wang et al., 1993), findings on predictors of students’ achievement and recent findings from systematic analyses on covariate selection in school VA models (Levy et al., 2020). More specifically, these results were obtained by using multilevel models and indicated that the inclusion of prior math achievement, prior language achievement, and covariates related to students’ sociodemographic and sociocultural backgrounds (i.e., socioeconomic status of the parents, languages spoken at home, migration status, and sex) into school VA models can make a difference in controlling for between-school differences in student intake and in the resulting school VA scores. Hence, these covariates were included into all statistical models in the present study. One limitation of the study by Levy et al. (2020) was that only one model type was used (i.e., multilevel model); here we contrast several model types.

We addressed two main research questions:

(1) How is the predictive power of school VA models (in predicting student academic scores) affected by different types of classic and more modern models?
(2) How sensitive is schools’ VA ranking to the selection of model types for the VA model?

## Materials and Methods

### Participants

This study is a secondary analysis and uses longitudinal large-scale data obtained from the Luxembourg School Monitoring Programme *ÉpStan* (LUCET, 2019). *ÉpStan* assesses students’ academic competencies (in math and languages), their subjective achievement motivation as well as information on their sociodemographic and sociocultural background at the beginning of the grade levels 1, 3, 5, 7, and 9. Every year, the entire student population in each of the concerned grade levels participates in the *ÉpStan*. In the present paper, we used longitudinal data from the student cohort that participated in *ÉpStan* in grade 1 in 2014.

For our analyses, we included only those *N* = 3,026 students attending 153 primary schools with complete cases on all variables (see Table 2 for details on sample composition and excluded students). Excluded students (*N* = 1,977) were either absent on the day of testing in third grade (*N* = 1,068; e.g., due to illness or grade repetition), or they changed schools between grades 1 and 3 (*N* = 332), or they had at least one missing value in the relevant covariates (*N = 577*). Excluded students had lower achievement values than included students, indicating among others that non-participation in grade 3 could most likely be due to repeating a grade between first and third grade. Treatment of missing data is a highly debated subject in many areas, also in VA research (e.g., Dearden et al., 2011). Here, we decided to analyze only complete cases, as the model comparisons would otherwise depend on assumptions made at the imputation process which could favor particular model types and hence prevent a clear interpretation of their results.

**TABLE 2:** Details on the sample composition and excluded students.

	Included students^a^	Excluded (at least one missing value)	Excluded (no participation in grade 3)	Excluded (students switched school)
Number of students	3,026	577	1,068	332
Mean prior math ach. in grade 1	523 (*SD* = 91)	503 (*SD* = 90)	437 (*SD* = 103)	499 (*SD* = 82)
Mean prior language ach. in grade 1	523 (*SD* = 92)	495 (*SD* = 97)	441 (*SD* = 103)	489 (*SD* = 85)
Percentage of female students	50	48	47	49
First language of instruction not spoken at home	49	58	65	60
Mean HISEI score	50.1 (*SD* = 15.4)	47.0 (*SD* = 15.0)	42.6 (*SD* = 15.5)	44.9 (*SD* = 15.2)
Mean math ach. in grade 3	519 (*SD* = 103)	495 (*SD* = 108)	–	479(*SD* = 93)
Mean language ach. in grade 3	518 (*SD* = 101)	482 (*SD* = 101)	–	474 (*SD* = 103)

*ach., achievement. ^*a*^Based on the criteria described above.*

The *ÉpStan* has a proper legal basis and the national committee for data protection gave its approval. Appropriate ethical standards were respected (American Psychological Association, 2017). All participating children and their parents or legal guardians were duly informed before the data collection, and had the possibility to opt-out. To ensure students’ privacy and in accordance with the European General Data Protection Regulation, collected data were pseudonymized with a so called “Trusted Third Party” (for more information see LUCET, 2019). For the present analysis, an anonymized dataset was used.

### Measures

#### Academic Achievement

VA modeling requires a choice of academic achievement as outcome measure, and often uses previous academic achievement as a covariate. Since for our data we have two equally appropriate choices—namely math and language achievement—we computed VA scores for both and report all results (for a recent meta-analysis on the mutual relationship between language and mathematics, see Peng et al., 2020). These two achievement measures from grade 3 were used as outcome variables, while the same scores from grade 1 were used as covariates (i.e., as a measure of prior achievement). At the very beginning of grades 1 and 3, all achievement measures were assessed with standardized achievement tests, which were developed on the basis of the national curriculum standards (defined by the Ministry of National Education, Children and Youth, 2011) by interdisciplinary expert groups, thus assuring content validity (Fischbach et al., 2014). The tests were administered in the classroom setting, given in a paper-and-pencil format, and mostly based on closed-format items. To scale the items, a unidimensional Rasch model was used (Fischbach et al., 2014; see Wu et al., 2007; Nagy and Neumann, 2010). Weighted likelihood estimates (WLE; Warm, 1989) were used as measures of students’ achievement (Fischbach et al., 2014). The reliability scores of all achievement scales were calculated using the function *WLErel* from the *TAM* package version 3.3.10 (Robitzsch et al., 2019), which estimates reliability scores based on WLE values and their standard errors.

##### Math achievement

*The math tests in grade 3* were constructed in German because the language of instruction in grades 1 and 2 is German. Math items assessed children’s competencies in three areas: “numbers and operations,” “space and form,” and “quantities and measures.” The reliability of the math test scores in grade 3 was 0.90. *Math achievement in grade 1* (i.e., prior math achievement) was assessed in Luxembourgish (which is, although politically and culturally a language on its own, linguistically speaking a variety of German, see Dalby, 1999) because the language of instruction in preschool is Luxembourgish. Mathematics items assessed children’s competencies in the domains “numbers and operations,” “space and shape,” and “size and measurement”^1^. The reliability of the math test scores in grade 1 was 0.75.

##### Language achievement

*Language achievement in grade 3* was operationalized by the children’s listening and reading comprehension in the German language. Listening comprehension was based on the subskills “identifying and applying information presented in a text” and “construing information and activating listening strategies.” Reading comprehension was assessed with the subskills “identifying and applying information presented in a text” and “construing information and activating reading strategies/techniques”^1^. The reliability of the listening comprehension and the reading comprehension test scores in grade 3 were 0.81 and 0.88, respectively. The correlation between those two achievement scores was 0.69. We computed a mean score across listening and reading comprehension in the German language to represent students’ language achievement in grade 3 in order to have only one dependent variable. *Language achievement in grade 1* (i.e., prior language achievement) consists of “early literacy comprehension” and “listening comprehension” in Luxembourgish in grade 1 because the language of instruction in preschool is Luxembourgish. Listening comprehension was assessed with the two subskills “identifying and applying information presented in a text” and “construing information and activating listening strategies” with different kinds of texts, which were played from an audio recording. Early literacy comprehension was assessed with the subskills “phonological awareness,” “visual discrimination,” and “understanding of the alphabetic principle”^2^. The reliability of the listening comprehension and the reading comprehension test scores in grade 1 were both 0.70. Contrary to the language achievement measures in grade three, both the listening and the reading score were included into the models instead of averaging them in order to keep as much information as possible, as these two scores only correlated with each other at 0.50.

##### A note on psychometric quality of achievement measures

The present study is a secondary analysis and relies on archive data for which only limited information on psychometric quality of achievement measures is available (see Appelbaum et al., 2018). Of note the underlying data are already used in real-life and drive political decisions and hence psychometric data quality has been optimized in that regard. As noted above, the present domain-specific tests were developed by expert panels (i.e., teachers, content-specialists on teaching and learning, psychometricians) to ensure content validity of all test items. All test items have also undergone intensive pilot-testing and psychometric quality checks concerning their empirical fit to the Rasch-Model that was used to derive WLE estimates representing students’ domain-specific achievements in grades 1 and 3. Further, all test items were examined whether they exhibit differential item functioning across student cohorts attending the same grade level to allow for commensurable measures across time. These psychometric quality measures helped to ensure structural validity of the test items within and across student cohorts. Additional analyses on their convergent and discriminant validity showed that domain-specific achievement test scores in both grade 1 and grade 3 followed the theoretically predicted pattern to academic self-concepts in matching and non-matching domains (Niepel et al., 2017; van der Westhuizen et al., 2019). Finally, the WLE-scores representing students’ domain-specific achievement demonstrated score reliability (with score reliability ranging between 0.70 and 0.90) that suffices research purposes (Schmitt, 1996).

#### Sociodemographic and Sociocultural Background Variables

To obtain information on children’s sociodemographic and sociocultural background, a parents’ questionnaire was administered in grade 1. Parents were asked to locate their profession within a given list of occupational categories (e.g., academia or craft); these categories were based on the ISCO classification (International Standard Classification of Occupations). For each occupational category, the average value of the ISEI scale, which is a validated scale (International Socio-Economic Index of occupational status, see Ganzeboom, 2010), was computed to obtain a proxy for the socioeconomic status of the parents (SES). In our grade 1 dataset, ISEI values have a mean of 50.1 and a standard deviation of 15.4. In the first PISA tests in 2000, the average ISEI for all OECD countries was 48.8 (OECD and UNESCO Institute for Statistics, 2003). Parents were also asked where they and their child were born to indicate their immigration status, resulting in the immigration status categories “native,” “first generation,” and “second generation.” In the present analyses, immigration status was coded as dummy variables with “native” being the reference category. In addition to the questionnaire filled out by the parents, grade 1 students also filled out a questionnaire on their own, where they were asked to indicate language(s) spoken with their father and their mother, respectively. As the first language of instruction is Luxembourgish, not speaking any Luxembourgish at home represents a challenge for the newly enrolled students. We thus created a dummy variable to differentiate between those students who do not speak any Luxembourgish at home and those who speak Luxembourgish with at least one parent (reference category). Students’ sex was retrieved from the official database of the Ministry of National Education, Children and Youth. Table 2 includes among others an overview of sociodemographic and sociocultural variables of all 3,026 students from 153 schools.

### Analysis

All analyses were conducted using R version 3.6.1 (R Core Team, 2019); the scripts can be found online at https://osf.io/rgt8x/?view_only=752453b81cd243e0b4ebfe33e1a74c33. In order to run models as similarly as possible, the *caret* package version 6.0.85 (Kuhn, 2019) was used as a wrapper of most functions. For all models except for the multilevel model, we followed the steps in Eqs 1 and 2 for prediction and VA estimation. Unless otherwise stated, the function call of the model was defined as follows:

Achievement_in_grade_3 ∼ Prior_Math_Achievement + Prior_Reading_Achievement + Prior_Language_Achievement + SES + migration_status + language_spoken_at_home + sex.

Hence, achievement in grade 3 is our outcome y variable with the others as covariates (see the *caret* package for details on function calls). Note that the “+” operator is treated as selecting covariates from the data, where the model type determines how the covariates are combined (e.g., for random forest covariates are selected to form tree branches, hence the “+” is not literal addition).

As is standard in machine learning, the dataset was randomly split into a *training-set* which contains 70% of the data and is used to fit or “train” models and a *test-set* which contains the remaining 30% of the data and which is used to evaluate the fitted model’s ability to predict new (“out-of-sample”) data (prediction accuracy was estimated via R-squared). To prevent the results from being dependent on a particular split of the data, the above procedure (i.e., split, train, test) was repeated 100 times, thus creating 100 *training*- and *test*-sets and the prediction performance averaged across those repetitions (100 was chosen to balance estimation with computing limitations). The parameter ranges we specified for hyper parameter selection (i.e., grid search) were selected for each parameter in order to span reasonable values. These are generally based on suggested default ranges for each model that come from standard practice, while respecting computational limitations. We report all values tested below. We performed cross-validation for hyper parameter selection, comparing the resampled results across models for the best performing hyper parameter set (for more detail on resampling procedures, see, e.g., Hothorn et al., 2005; Eugster and Leisch, 2011). For models with no hyper parameters (e.g., linear regression) we performed the identical cross-validation resampling procedure for between model comparison. Model performance was then compared on the resampled results.

#### Model Comparison

Fundamentally, when creating a predictive model of the form *y = f(x)*, both statistics and machine learning practitioners would specify a function space to optimize over (e.g., polynomials) given some loss criterion (e.g., mean-squared error). Where they differ is in these details of function space, criteria, and fitting procedure. Table 1 depicts an overview of the different model types used, which relationship they assume between dependent and independent variable(s), some specific criteria to each model type, which package and function was used, and which hyper parameters were defined. We use common models for prediction, including ensemble approaches (random forest and boosting) which combine across many weaker models to improve performance, and general function fitting models (neural networks and support vector machines) which transform the inputs into a potentially more useful space for prediction. A more detailed conceptual description and implementation can be found in the Online Supplement A1.

#### Estimation of VA Scores

For most statistical models used, school VA scores were calculated as the mean difference between the actual and the predicted achievement values from each student in a certain school (i.e., the residuals).

The only exception were multilevel models, where the VA score of a school was quantified in terms of an estimate of the random effect for a particular school at school level (i.e., the residual of a certain school; see Ferrão and Goldstein, 2009). School VA scores were thus estimated using the *ranef* function from the *lme4* package (Bates et al., 2015). Note that this is only the case for the VA score; the resampling results were estimated the same way across all models.

#### Operationalization of the Research Questions

To address Research Question 1, we evaluated the predictive power of the underlying VA model in terms of the total amount of variance (*R*^2^s) explained. This was estimated with the *resamples* function from the *caret* package (Kuhn, 2019) by the same estimation process for all models used (i.e., based on the comparison between predicted and observed values of student achievement, using the model’s *predict* function).

Further, we tackled Research Question 2 on how the VA ranking of schools depends on the model selection by computing correlations of school VA scores with each other as obtained from various school VA models and by analyzing the implications of model selection on benchmark classifications. Specifically, following current benchmarks (e.g., Marzano and Toth, 2013), we classified the best 25% of schools (in terms of VA scores) as “highly effective,” the worst 25% as “needs improvement,” and the remaining 50% of schools (i.e., between the 25^th^ and 75^th^ percentiles) as “moderately effective.” For every school VA model, we computed the percentage of disagreements by calculating the percentage of schools identified at a different benchmark classification as the one resulting from the multilevel model, which is one of the two most commonly used school VA models and which in this analysis serves as a reference (Kurtz, 2018; Levy et al., 2019). More concretely, to get the percentage of disagreements, for every model, the number of schools ranking at a different benchmark than by the multilevel model was divided by the total number of schools and multiplied by 100. Smaller values represent a higher concordance with the benchmark classifications from the multilevel model; higher values indicate a higher rate of disagreements. While all preceding operationalizations include results from all 153 schools, real-life implications of benchmark classifications based on different model types will be illustrated based on five example schools.

## Results

### Research Question 1: How Is the Predictive Power of School VA Models Affected by Different Types of Classic and More Modern Models?

#### School VA Models for Math Achievement

**FIGURE 1:**
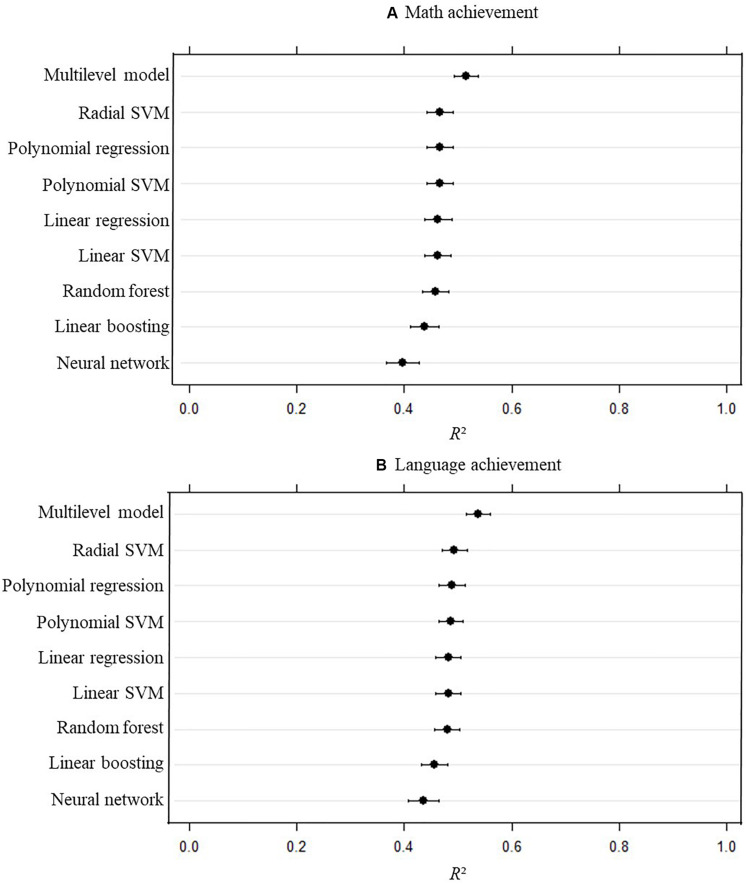
Amount of explained variance (*R*^2^) for each statistical model with math achievement **(A)** and language achievement **(B)** as a dependent variable with confidence intervals computed on the out-of-sample measures obtained via the 100 repetitions. Error bars represent 95% confidence intervals computed on the out-of-sample measures obtained via the 100 repetitions. SVM, support vector machines.

Figure 1A shows the mean and the confidence intervals of the amount of explained variance (*R*^2^) for the 100 cross-validations of each statistical model with math achievement as a dependent variable. It can be observed that the values of the different models are close to each other, with the highest predictive power error for the multilevel model (mean *R*^2^ of 0.51) and the lowest for neural networks (mean *R*^2^ of 0.40). For all the other models, the mean *R*^2^ was between 0.44 and 0.47.

#### School VA Models for Language Achievement

Figure 1B shows the confidence intervals of explained variance (*R*^2^) for the 100 cross-validations of each statistical model with language achievement as a dependent variable. The results are analogously to the school VA models for math. It can be observed that the values of the different models are close to each other, with the highest predictive power for the multilevel model (mean *R*^2^ of 0.54) and the lowest mean *R*^2^ for neural networks (0.44) and linear boosting (0.46). For all the other models, the mean *R*^2^ was between 0.48 and 0.49.

### Research Question 2: How Sensitive Is Schools’ VA Ranking to the Selection of Statistical Models Types for the VA Model?

#### Correlations Between School VA Scores by Model Type

##### School VA models for math achievement

Table 3 shows the correlations between the school VA scores resulting from the VA models for math achievement based on different statistical models. They range between 0.88 and 1.00 (*Mdn* = 0.98). The lowest correlations can be observed for those school VA scores resulting from linear boosting (ranging from 0.88 to 0.94) and neural networks (ranging from 0.94 to 0.97). For all other model types, the resulting school VA scores correlate with each other to at least *r* = 0.98.

**TABLE 3:** Correlations between school VA scores resulting from different model types with math achievement as a dependent variable.

Model type	Linear regression	Multilevel model	Polynomial regression	Random forests	Neural networks	Linear SVM	Poly-nomial SVM	Radial SVM
Linear regression	–							
Multilevel model	0.98	–						
Polynomial regression	1.00	0.98	–					
Random forests	0.99	0.98	0.99	–				
Neural networks	0.96	0.94	0.96	0.97	–			
Linear SVM	1.00	0.98	1.00	0.99	0.96	–		
Polynomial SVM	1.00	0.98	1.00	0.99	0.96	1.00	–	
Radial SVM	1.00	0.98	1.00	0.99	0.96	1.00	1.00	–
Linear boosting	0.93	0.92	0.93	0.94	0.88	0.93	0.93	0.93

*SVM, support vector machines. All correlations are significant with *p* < 0.01.*

##### School VA models for language achievement

A similar pattern can be observed for school VA models for language achievement (Table 4). Correlations between the resulting school VA scores range between 0.89 and 1.00 (*Mdn* = 0.98). The lowest correlations can be observed for those school VA scores resulting from linear boosting (ranging from 0.89 to 0.97) and neural networks (ranging from 0.92 to 0.94). For all other model types, the resulting school VA scores correlate with each other to at least *r* = 0.95.

**TABLE 4:** Correlations between school VA scores resulting from different model types with language achievement as a dependent variable.

Model type	Linear regression	Multilevel model	Polynomial regression	Random forests	Neural networks	Linear SVM	Poly-nomial SVM	Radial SVM
Linear regression	–							
Multilevel model	0.97	–						
Polynomial regression	1.00	0.96	–					
Random forests	0.99	0.95	0.99	–				
Neural networks	0.93	0.93	0.92	0.94	–			
Linear SVM	1.00	0.97	1.00	0.99	0.93	–		
Polynomial SVM	1.00	0.96	1.00	0.99	0.93	1.00	–	
Radial SVM	0.99	0.96	1.00	0.99	0.94	0.99	1.00	–
Linear boosting	0.96	0.92	0.96	0.97	0.89	0.96	0.96	0.96

*SVM, support vector machines. All correlations are significant with *p* < 0.01.*

#### Percentage of Disagreement in Comparison to the Multilevel Model

In the following section, we evaluate to what extent the classification of schools into one of the three benchmark classifications “needs improvement,” “moderately effective,” and “highly effective” depends on the particular model used to compute the VA scores. More specifically, we will use the classification that results from the multilevel model as the reference against which to compare the classifications that results from all other VA scores estimation methods.

##### School VA models for math achievement

**FIGURE 2:**
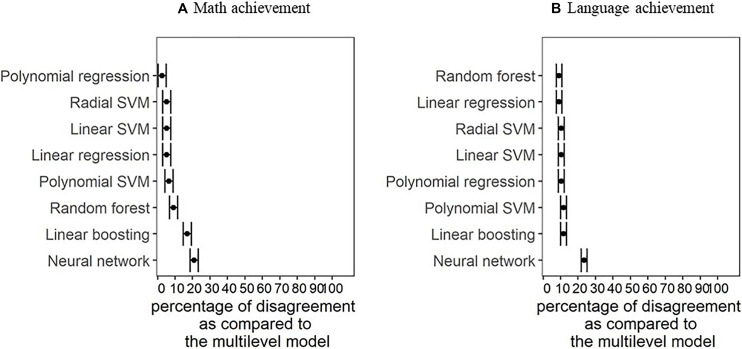
Percentage of disagreement as compared to the benchmark reached based on VA scores from the multilevel model with math achievement **(A)** and language achievement **(B)** as a dependent variable. SVM, support vector machines.

Figure 2 shows the percentage of disagreement as compared to the school VA scores based on the multilevel model. In Figure 2A, representing the school VA models with math achievement as a dependent variable, it can be observed that for most statistical models, the percentage of disagreement is under 10%. The only exceptions are school VA scores based on the neural network model (21% of disagreements) and the linear boosting model (17% of disagreements). A detailed overview of percentages of disagreement from school VA models for math achievement compared to those from the multilevel model can be found in Table 5.

**TABLE 5:** Percentage of disagreement as compared to the benchmark reached based on VA scores from the multilevel model with math achievement as a dependent variable.

	Classified by multilevel model as:			
Model	Needs improvement^a^	Moderately effective^b^	Highly effective^c^	Total percentage of disagreement
Linear regression	7.89	5.19	2.63	5.23
Polynomial regression	2.63	2.60	2.63	2.61
Random forest	7.89	9.09	10.53	9.15
Neural network	23.68	20.78	18.42	20.92
Linear boosting	10.53	16.88	23.68	16.99
Linear SVM^d^	7.89	5.19	2.63	5.23
Polynomial SVM^d^	8.89	6.49	5.26	6.54
Radial SVM^d^	2.63	5.19	7.89	5.23

*^*a*^Below the 25^*th*^ percentile. ^*b*^Between the 25^*th*^ and the 75^*th*^ percentiles. ^*c*^Above the 75^*th*^ percentile. ^*d*^SVM, support vector machines.*

##### School VA models for language achievement

Figure 2B shows how many schools’ benchmark classifications would be in disagreement based on their language VA scores resulting from the different statistical models. Analogously to the results for the school VA models for math achievement, it can be observed that the percentages of disagreement of most statistical models are similar to each other. More specifically, the percentages of disagreement are around 10% for all models except for the neural network (24%). A detailed overview of percentages of disagreement from school VA models for language achievement compared to those from the multilevel model can be found in Table 6.

**TABLE 6:** Percentage of disagreement as compared to the benchmark reached based on VA scores from the multilevel model with language achievement as a dependent variable.

	Classified by multilevel model as:			
Model	Needs improvement^a^	Moderately effective^b^	Highly effective^c^	Total percentage of disagreement
Linear regression	7.89	9.09	10.53	9.15
Polynomial regression	7.89	10.39	13.16	10.46
Random forest	5.26	9.09	13.16	9.15
Neural network	21.05	23.38	26.32	23.53
Linear boosting	13.16	11.69	10.53	11.76
Linear SVM^d^	7.89	10.39	13.16	10.46
Polynomial SVM^d^	7.89	11.69	15.79	11.76
Radial SVM^d^	5.26	10.39	15.79	10.46

*^*a*^Below the 25^*th*^ percentile. ^*b*^Between the 25^*th*^ and the 75^*th*^ percentiles. ^*c*^Above the 75^*th*^ percentile. ^*d*^SVM, support vector machines.*

#### Real-Life Implications on the Example of Five Schools

**FIGURE 3:**
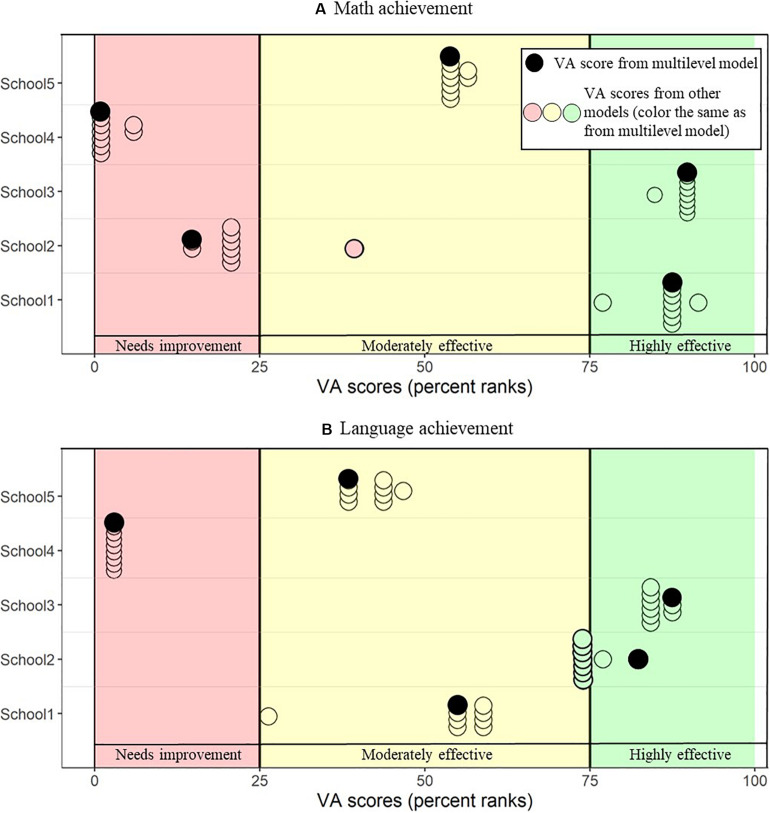
Range of percentiles resulting from math **(A)** and language **(B)** VA scores for five example schools. Every dot represents the school VA percentile as obtained from a certain VA model. At the 25^th^ and 75^th^ percentiles, there are cut-off lines to define the border between schools classified as “needs improvement” (colored in red), “moderately effective” (colored in yellow), and “highly effective” (colored in green). VA percentiles resulting from the multilevel model are marked in black. All the other models are represented by dots in the same color as the area the VA percentile from the multilevel is situated, allowing to see how often schools’ classifications would be in disagreement.

Figure 3 illustrates the real-life implications that the use of different statistical models for the estimation of VA scores may have for five schools that were chosen as examples (see Table 7 for descriptive data on these schools; these are the same schools that were presented in Levy et al., 2020). It shows the range of the VA percentiles resulting from the different statistical models for these schools and illustrates that, despite high correlations across schools, there is variation within individual schools. More specifically, for most schools, when comparing schools’ VA percentiles within the same dependent variable, schools would be categorized within the same benchmark (i.e., constantly within “needs improvement,” “moderately effective,” and “highly effective”), regardless of the statistical model used (the exact values can be found in Table 8). However, for school 2, depending on the type of model used, the school is classified differently. Interestingly, for every school except for school 1 the most extreme values of VA percentiles (i.e., highest or lowest) are reached with the multilevel as the underlying school VA model.

**TABLE 7:** Descriptive data from the five example schools shown in Figure 3.

	School 1	School 2	School 3	School 4	School 5
Number of students	18	52	33	49	26
Mean prior math achievement in grade 1	459	575	509	592	534
Mean prior language achievement in grade 1	468	567	511	587	506
Percentage of female students	33%	54%	61%	53%	58%
Percentage of “First language of instruction not spoken at home”	66	33	55	80	46
Mean HISEI score	41.0	56.2	54.6	56.8	48.2
Mean math achievement in grade 3	504	540	543	498	517
Mean language achievement in grade 3	476	579	527	470	510

**TABLE 8:** Percentiles resulting from different VA models for the five example schools.

	School 1		School 2		School 3		School 4		School 5	
	Math	Language	Math	Language	Math	Language	Math	Language	Math	Language
Linear regression	88	55	21	77	89	86	1	2	54	39
Multilevel model	88	53	14	82	91	88	0	1	53	39
Polynomial regression	88	57	20	75	90	85	1	2	57	43
Random forest	87	59	19	72	89	83	2	2	55	42
Neural network	77	26	39	75	85	84	5	5	55	45
Linear boosting	91	57	16	76	89	85	7	3	55	47
Linear SVM	88	57	20	74	89	87	1	2	53	38
Polynomial SVM	88	61	22	74	90	88	1	2	55	39
Radial SVM	88	56	22	74	90	86	1	2	57	44

*SVM, support vector machines.*

## Discussion

School VA models are statistical models designed to estimate school effectiveness (i.e., school VA scores) based on the evolution of students’ achievement. These VA scores are often used for accountability and high-stakes decisions to allocate financial or personal resources to schools. However, despite their practical relevance, there is currently no consensus on how to best estimate VA scores. The two most commonly used statistical models are linear regression and multilevel models (Kurtz, 2018; Levy et al., 2019) and some researchers have applied non-linear models for the estimation of school VA scores (Fitz-Gibbon, 1997; Lopez-Martin et al., 2014). An alternative approach to these classical models involves machine learning methods, which social sciences are drawing on more as larger and more complex data sets become increasingly available, as new types of data require new data analytical approaches. Such techniques have already evolved in fields with a long tradition in crunching big data (e.g., gene technology). One reason to investigate the use of machine learning methods for the estimation of VA scores is because they can take complex interactions between students’ intake characteristics as well as complex functional forms how these characteristics are related to outcome variables into account. This is different to the classic approach of how VA scores are computed where typically only a linear function is specified that relates the pretest measure (and perhaps further covariates) to the outcome measure. Our goal in the present paper was to contribute to the discussion on how best to compute VA scores while at the same time evaluating the potential of modern machine learning methods in this discipline. More specifically, the present study aimed to address two main research questions:

(1) How is the predictive power of school VA models affected by different types of classic and more modern models?
(2) How sensitive is schools’ VA ranking to the selection of model types for the VA model?

In the following, we discuss the implications from the results of the math and language school VA models together because the focus of the present paper is on the model choice rather than on the domain of the dependent variable. The findings are consistent across both domains, which suggests that they are robust and that the results from of the math and language school VA models can be grouped together in the discussion.

### How Is the Predictive Power of School VA Models Affected by Different Types of Classic and More Modern Models?

The predictive power of school VA models was very similar for most model types. The only exceptions were the values from the multilevel model and from the neural network, which were significantly different from the other model types. More concretely, the multilevel model performed better than all the other models and the neural network worse. These findings were consistent across dependent variables (i.e., math achievement and language achievement).

The fact that most of the machine learning models were not significantly different from the linear, and the multilevel model outperforming, might seem surprising on the first sight. However, this is likely due to a few reasons. One is a simple model complexity tradeoff, and that out-of-sample performance penalizes overfitting the training data. The performance of these data driven approaches is always subject to basic statistical issues of model complexity and bias-variance tradeoffs (e.g., Hastie et al., 2009). Models with low complexity (e.g., low number of parameters) can perform better for out of sample (or “test”) datasets, as complex models are prone to overfit (i.e., adjust the model to noise). While educational domains are likely highly structured, it is not *a priori* obvious if atheoretical non-linear or complex models will be able to capture this structure. Without formal theory that makes strong predictions in this domain, we must rely on statistical comparisons to select the machine learning models for estimating VA scores. Either the non-linear structure is not appropriate for the data (hence no benefit beyond a linear equation) or the noise in data is high enough to prefer the simpler models.

Additionally, the multilevel model’s performance could be explained by the fact it took into account the nested structure of the data, which the other models did not. This indicates that fitting VA models only across schools is not enough, as there seems to be important information within schools that can add to explaining variance. Of course, it could be argued that the multilevel models received more information than the other models, as schoolID was added to the equation. However, the standard logic of VA estimation means such information cannot be appropriately included, unless one can estimate school-level error independent of non-school factors (which the multilevel model allows). They are the only one of the model types chosen for the present analysis that is able to take into account the nested structure of the data appropriately. Just adding school as a covariate to the other models would thus not contribute to a solution, as this would result in breaking the logic in estimation of VA scores, as they are calculated for schools without school information.

As opposed to the findings from Schiltz et al. (2018), the predictive power of the linear regression was not significantly different from the random forest. This could be due to differences in the datasets: Schiltz et al. (2018) used Italian population data, while we used Luxembourgish population data. Additionally, Schiltz et al. (2018) used secondary school students from Italian population data, while we used primary school students from Luxembourgish population data. These differences suggest that model performance can depend on the dataset used, and that caution should be given in generalizing beyond one dataset. Additionally, different covariates were included, such as our inclusion of language(s) spoken at home, a significant variable for the Luxembourgish population (e.g., Ugen et al., 2013; Martini and Ugen, 2018). Given our previous results (Levy et al., 2020), covariate choice is highly important in model performance, and therefore a critical concern in comparing different datasets.

However, the present results of the superior performance of multilevel models offer a suggestion on default model choice. The exploration of school VA scores on primary school aged children is especially relevant in heterogeneous populations such as Luxembourg, as socioeconomic disparities appear already within the two first grades of primary school (Hoffmann et al., 2018). For this specific context, multilevel models outperform classical linear and polynomial regressions, as well as different machine learning models. While in many domains linear regression is widely accepted as the default model, changing this default to multilevel modeling works well for hierarchically structured data (as discussed by McElreath, 2017). This seems to be the case for school VA models, as well. While this is sensible since the data clearly have a hierarchical structure (e.g., students nested within schools); the present results statistically demonstrate the multilevel model’s performance.

### How Sensitive Is Schools’ VA Ranking to the Selection of Model Types for the VA Model?

School VA scores resulting from the different model types correlated highly with each other (ranging from 0.88 to 1.00 for school VA models in math and from 0.89 to 1.00 for school VA models in language). At first glance, this might suggest that the resulting school VA scores are similar to each other across schools, which could even lead to the—premature—conclusion that the least complex model, in terms of parsimony and transparency (i.e., the linear regression because of its intuitive interpretability, see, e.g., Molnar, 2020) should be chosen (see e.g., Cohen, 1990; Wilkinson and Task Force on Statistical Inference, 1999). However, high correlations between different school VA scores will not necessarily prevent disagreements of classifications from individual schools (e.g., Timmermans et al., 2011; Ehlert et al., 2014; Levy et al., 2020). This is why, in a second step, the school VA scores were transferred to percentiles and then benchmarks were used to classify schools (i.e., “needs improvement,” “moderately effective,” and “highly effective”).

We compared the resulting benchmarks from all models to those obtained by the multilevel model by calculating the percentage of disagreement. The percentage of disagreement was mostly around 10%. The only exceptions were neural network for school VA scores in math and language and linear boosting for school VA scores in language. However, these two model types were also the ones with the lowest predictive power and it is thus not surprising that their resulting benchmark classifications deviate the most.

As for all the other models, the percentage of disagreement seems low. However, 10% of disagreement means that for most models, at least 15 out of these 153 schools would be classified differently if another model than multilevel models is used (assuming multilevel models provide the reference classification). Given that these benchmark classifications can have high-stakes consequences, the present results underline the relevance of model choice, as individual schools’ VA rankings are sensitive to the selection of model types. To further illustrate the real-life implications the model selection can have on individual schools, we will discuss the example of five schools.

### Real-Life Implications

Despite very high correlations between school VA scores across models, we can still see differences in benchmark classifications for some of our example schools depending on the model used. This raises the question “how high should a correlation be for it not to matter?” This question cannot be answered in a general way, as it depends on the very practical and political issue of how these VA scores are used in practice. Rather, it should be kept in mind by any researcher, practitioner, or politician when applying or interpreting results from school VA models. Most importantly, it should be kept in mind, especially when taking high-stakes or accountability decisions based on VA models, that any single value used to evaluate schools’ effectiveness represents only one possible truth; a single point estimate. One alternative would be to include confidence intervals on VA score rankings and classifications, as well as a combination of interdisciplinary methods as was done in the present study. This would allow a range of possible VA scores for every school VA scores, incorporating uncertainty underlying the estimate. However, ethical implications of the results from the present study and of the use of machine learning methods for consequential decisions in a discipline they were not specifically designed for (i.e., education) should be discussed.

### Ethical Implications

The idea of using machine learning to make better and more objective predictions than with conventional statistical methods sounds promising for the application on school VA models, and ideas on how to use machine learning methods for education have been around for decades (Romero and Ventura, 2007, 2010, 2020). However, even though machine learning techniques have driven progress in numerous other disciplines, such as automated face identification (Taigman et al., 2014) or beating human players at the game Go (Silver et al., 2017), their potential downsides and limits need to be discussed, too (see, e.g., Synced, 2018). For example, one study on the diagnostic analysis of medical images reported that out of 516 studies, only 6% tested their algorithms on datasets in different hospitals (Kim et al., 2019). This can result in false associations, such as the association between images from portable x-ray machines and illness (as described by Couzin-Frankel, 2019). This happened because these portable x-ray machines were only used when the patients were already too ill to get out of their bed and as images from these x-ray machines look different from the ones when a patient is not lying down and was thus a circular conclusion. This shows how important data collection processes are, as biased data will lead to biased results, regardless of the applied model.

Image recognition has the advantage that it is still comparably easy for humans to objectively judge whether the classification done by a certain algorithm is true or not. However, this becomes more challenging for concepts such as school VA scores, where the entire point is that we do not know schools’ effectiveness, which is why we are estimating VA models in the first place, in order to approximate a measure of schools’ effectiveness. Model performance is always limited by the model’s assumptions and the data used to train it (e.g., Hastie et al., 2009; Alpaydin and Bach, 2014). This highlights the importance of transparency and clear communication in how these models are estimated, selected, and used, as is also underlined by recent lawsuits (Paige, 2020; Paige and Amrein-Beardsley, 2020).

### Implications for Educational Practice

As discussed, a core concern with decisions based on estimates of VA scores is how to appropriately communicate limitations to stakeholders. For example, even if there were no differences across schools in “real” VA scores, a ranking of schools can still be constructed (based purely on randomness). These concerns, along with others presented above, leads to the suggestion that models used for the estimation of school VA scores should never be used alone for high-stakes decisions. This has been elaborated on a more general level in Barocas et al. (2019), where the authors stress the importance of the complementary use of these models together with observational, qualitative, and/or ethnographic studies. This goes in line with researchers recommending a combination of VA scores and observations for high-stakes decisions (e.g., Bacher-Hicks et al., 2019) or of using school VA scores only for informative purposes rather than accountability (e.g., Leckie and Goldstein, 2019). Additionally, even though multilevel models provided the best predictive power within the present dataset, this finding may not generalize to other contexts. We thus recommend that practitioners do not just implement the model suggested by the field, but instead follow a process for model selection with different model types which combine the expertise from different disciplines, as it has been done in the present study. Future work should develop standardized processes and benchmarks, such as following those from Hothorn et al. (2005) and Eugster and Leisch (2011). Optimally, a transparent process for model selection with different model types, combining expertise from multiple disciplines, should be implemented for the estimation of VA scores.

### Limitations and Future Work

Treatment of missing data is a highly discussed subject in many areas, also in VA research (e.g., Dearden et al., 2011). We decided to analyze only complete cases, as the model comparisons would otherwise already depend on assumptions made at the imputation process and could lead to differences in VA scores. For future research, it would be interesting to include those with missing cases, possibly comparing different imputation methods and/or by dummy coding whether an entry is missing or not. However, this should be done after the principle differences between different model types have been investigated, hence the importance of the present study.

School VA scores were computed differently in multilevel models as compared to the other model types (i.e., estimated based on the random effects at school level). On the one hand, this might make the comparison between the resulting VA scores unfair in favor of multilevel models. On the other hand, the amount of explained variance was estimated in the same way for all model types. Additionally, this estimation of VA scores by the random effects at school level is specifically how school VA scores are estimated in most cases, thus representing a realistic representation of practice (Ferrão and Goldstein, 2009; Levy et al., 2019). Furthermore, in most other studies comparing classical and machine learning approaches, machine learning approaches have an advantage due to less strict assumptions. More specifically, it is not possible to get a comparison that is fair in every aspect. However, we tried to keep as many aspects constant across model as possible.

Furthermore, the data was obtained with pen and paper rather than using a computer. The latter would have allowed to compute response time and avoid transcription errors. However, particular steps were taken to maximize objectivity and consistency, for example by double coding a random set of answers (Fischbach et al., 2014). However, other measures such as discriminant of convergent validity do not exist, yet. Future studies should thus investigate these important quality criteria, for example by matching the achievement test results with school grades. Given that the present study is a secondary analysis and relies on archive data, only limited information on the psychometric quality of achievement test scores was available and presented here (see Appelbaum et al., 2018). Of note, the domain-specific achievement tests that we used in the present study were developed to support their use for real-life and political decisions in educational settings in Luxembourg. The achievement tests also demonstrated score reliability that is typically considered to suffice research purposes (see Schmitt, 1996). Nevertheless, future work should consider whether the underlying reliability of the measures has an impact on model selection.

Additionally, as the present data set was already prepared (and thus simplified) by classical psychometric methods (i.e., IRT and WLE), it would be interesting for future works to compare the different models when using the raw data instead, as it could be that the machine learning models can make use of the higher level of complexity in the data.

The Luxembourgish school system consists of learning cycles, which usually take 2 years but can be extended to 3 years. Thus, the number of students who took part in grade 1 in 2014 but not in grade 3 in 2016 was quite high. This can introduce biases into our dataset, since the excluded students had lower achievement, lower socioeconomic status values, and a higher percentage of students who did not speak the first language of instruction at home than those who met the inclusion criteria. However, this problem exists in most educational datasets, as students who switched schools or repeated a grade are generally excluded from VA models in prior research; given the VA estimates are typically used for accountability purposes. This means that our data and results largely reflect the reality of how VA scores are typically estimated. Additionally, biases in the dataset will impact any model trained on that dataset similarly.

As previously discussed, the dataset is important in generalizing claims about model performance. Luxembourg is a particularly diverse and multilingual educational context compared to other school systems. Additionally, most applications of VA models estimate performance based on a 1-year time difference, while for us the difference between time points was 2 years (representing one learning cycle in the Luxembourg school system). Future research should replicate the present study to more homogeneous settings, and longitudinal data sets with 1 year, to determine to what degree our results are specific to the particular setting in Luxembourg.

The present study only used data from a single student cohort to obtain school VA score estimates. However, previous research suggests there is a naturally high variability in VA scores across cohorts (e.g., Sass, 2008; Newton et al., 2010; Minaya and Agasisti, 2019). Future research could thus extend the present study by including school VA scores obtained for several student cohorts to investigate whether there are schools with stable VA scores across cohorts/time within (or across) models and the extent to which the stability across cohorts is related to model selection.

While future work might be in creation of theory-driven models rather than a more explorative use of machine learning approaches, we were most interested in comparing standard approaches (both from machine learning and VA models). Our approach thus provides a relevant first step in extending existing research on the estimation of school VA models by investigating all those different approaches that are typically used to estimate school VA scores in particular or to deal with big amounts of data in general. As the multilevel model outperformed any of the standard machine learning approaches used, future research might expand the present study by considering machine learning models with a hierarchical structure that respect the logic underlying VA estimate.

## Conclusion

The present study investigated different statistical models for the estimation of school VA scores, finding that multilevel models outperformed classical linear and polynomial regressions, as well as a selective sample of different machine learning models. Even though the estimated VA scores from different model types correlated highly across schools, the percentage of disagreement as compared to benchmark classifications based on the multilevel model was substantial. Additionally, real-life implications for individual schools may be consequential depending on the model type used. Based on the present dataset, multilevel models would be recommended for the estimation of school VA scores because these models provide the most accurate predictions of student’s achievement. Also, because we observe that VA scores vary depending on specific model choices, we suggest that school VA scores should not be used as the only measure for accountability or high-stakes decisions and that they always be presented with confidence intervals. Optimally, a transparent process for model selection with different model types, combining expertise from multiple disciplines, should be implemented for the estimation of VA scores.

## Data Availability Statement

The data analyzed in this study is subject to the following licenses/restrictions: Sensible educational policy data from the Luxembourg School Monitoring Programme “Épreuves Standardisées” (www.epstan.lu) that has been kindly made available for this specific secondary analysis. Requests to access these datasets should be directed to www.epstan.lu.

## Ethics Statement

Ethical review and approval was not required for the study on human participants in accordance with the local legislation and institutional requirements. Written informed consent for participation was not provided by the participants’ legal guardians/next of kin because the present study is based on existing data. The existing dataset (ÉpStan) has a proper legal basis and has been approved by the national committee for data protection. Appropriate ethical standards were respected (American Psychological Association, 2017). All participating children and their parents or legal guardians were duly informed before the data collection, and had the possibility to opt-out.

## Author Contributions

All authors listed have made a substantial, direct and intellectual contribution to the work, and approved it for publication.

## Conflict of Interest

The authors declare that the research was conducted in the absence of any commercial or financial relationships that could be construed as a potential conflict of interest.

## Footnotes

1 https://epstan.lu/en/assessed-competences-31/

2 https://epstan.lu/en/assessed-competences-21/
