# Supplementary material for: Contrasting Classical and Machine Learning Approaches in the Estimation of Value-Added Scores in Large-Scale Educational Data
Source: Front Psychol. 2020 Aug 21;11:2190. doi: 10.3389/fpsyg.2020.02190 (PMC7472739; doi:10.3389/fpsyg.2020.02190)
Supplement: Supplementary file 1 [file Data_Sheet_1.docx]

# **A1. Conceptual description and implementation of the different model types**

### Classical approaches

Linear regression models. Linear regression models, including simple and multiple linear regression models, assume a linear relationship between the dependent and independent variable(s). Interestingly, it is quite difficult to make up a theoretical argument that the functional form needs to be strictly linear. Rather, most models of school learning implicitly draw on this assumption, and perhaps justify it saying that a linear function is parsimonious.

For the estimation of school VA scores, the dependent variable consists of an achievement measure for each student and the independent variables are student (background) variables (e.g., prior achievement). A certain school’s VA score is defined as the average residual across all students in this specific school.

Multilevel models. In multilevel models, also known as hierarchical linear models, mixed models, or random coefficient models (McNeish et al., 2017), the nested structure of the data is taken into account. Such a nested structure (i.e., the fact that students are nested in schools, meaning that students within the same school tend to be more similar to each other than students from different schools) is typical in the field of VA modeling (e.g., students nested in schools).

For the estimation of school VA scores, the dependent variable is an achievement measure and the independent variables are student (background) variables, same as the linear regression model. The largest difference from a linear regression model is that multilevel models directly take into account the hierarchical structure of the data and thus have multiple error terms. For the VA score of a certain school this means that it can be quantified in terms of an estimate of the residuals for this particular school at school level (see Ferrão & Goldstein, 2009), rather than estimated from student level predictions. The multilevel model is also different from other models in the sense that information on the school (e.g., the simplest form, the school ID) can (and has to) be included, as multilevel models are able to take into account this hierarchical structure in meaningful way without violating the core logic of VA estimation.

Polynomial regression models. Polynomial regression models follow the same logic as linear regression models, with the exception that they do not assume a linear relationship between the dependent and independent variable(s)(for more explanations and an illustration see, e.g., Edwards & Parry, 1993). A certain school’s VA score is also for polynomial regression models defined as the average residual across all students in this specific school.

### Machine learning approaches

Machine learning covers a large space of possible models to use, however our primary interest is in a set of data driven predictive models that expand on the basic linear model initially used. We selected models based on both previous use in the VA literature (Schiltz et al., 2018), and standard machine learning approaches commonly used in practice (e.g., Hellas et al., 2018). This included standard models suggested by introductory machine learning and data science textbooks (e.g., Alpaydin & Bach, 2014; Hastie et al., 2009), popular models used for machine learning competitions (e.g., kaggle, https://www.kaggle.com), and suggestions from expert practitioners (where most practitioners have a standard set of first pass "predictive models" they employ). Additionally, we wanted to draw from major classes of predictive models; nonlinear models, nonparametric models), and ensemble approaches (where multiple models are combined to predict outcomes). Here we briefly describe each approach used (for more detail see the two following books, which are available for free online: for a practical introduction see Boehmke & Greenwell, 2019; for a more technical background see Hastie et al., 2009).

Random Forest. Random forest models are one of the more common “off the shelf” methods used in machine learning (Breiman, 2001; Ziegler & König, 2014). They are an extension of decision trees models. A single decision tree model predicts the output y by creating a tree that sequentially partitions (splits) the input space, with each “leaf” the tree produces a new split that follows a simple rule (e.g., IF x_1_ > 5, then follow left branch, ELSE follow right branch). These splits allow for nonlinearities in predicting the output space. The final output at each leaf is then predicted via a simple rule, such as a constant value for each leaf. While individual decision trees are prone to overfitting, random forests overcome this through combining many simpler models. Random forests fit a large amount of single decision trees to the data, and combine them by averaging their predicted output. When fitting these individual models, random forests sample a subset of the training data, with the goal of making many uncorrelated models to combine (Ziegler & König, 2014). Various methods can be used to decide when to split individual trees or construct a new one (Breiman, 2001; Ziegler & König, 2014).

Neural networks. Artificial neural networks, while initially inspired by simple models of biological neurons, have become standard in machine learning as general function approximators (Goodfellow et al., 2016). The simple feedforward neural net can also be understood as a sequential logistic regression, where inputs are passed through a series of linear weights and nonlinear equations (e.g., a sigmoid) to predict the output. These “multiple layers” allow neural nets to approximate most nonlinear functions, by essentially performing feature selection before prediction. However, these multiple layers can also overfit, which can lead to poorer out of sample performance. They can also be thought of as a soft version of a decision tree (Frosst & Hinton, 2017), where the nonlinearity produces a graded and weighted split of the input space.

Support vector machines. Support vector machines (SVM) are extensions of regression approaches that take advantage of two major methods: finding the minimal margin hyperplane based on “support vectors” (i.e., exemplar data points), along with the kernel method (see, e.g., Hastie et al., 2009). Support vector regression attempts to fit a hyperplane to data, much like standard regression, while minimizing the margin or distance between support vectors. This acts as a constrained regression, as the SVM is attempting to span as much data as possible with a small margin (for an illustrated example see Awad & Khanna, 2015).

In order to incorporate nonlinearities, SVM also uses the kernel method (aka the kernel “trick”). One way of incorporating nonlinearities is first performing a nonlinear transformation of the data and then learn a simple linear function on the transformed data. The kernel method allows SVMs to do this without performing the often computationally expensive initial transformation, by employing kernel functions in their fitting procedure. Kernel functions are measures of similarity between data points, but are equivalent to dot products in some transformed space. Various kernel functions exist, including linear (i.e., standard dot product), polynomial, and radial basis functions.

Boosting. Boosting methods are a type of ensemble method, that trains multiple weaker models and combines performance across them (Friedman, 2001), similar to random forests. However, boosting methods also sequentially train models based on performance of past models. In particular, the gradient boosting algorithm trains new models using the residuals of the previously trained model, attempting to perform where previous models fail. Final performance is then predicted based on a weighted average of many of these simpler models. Much like random forests, boosting is a common "off the shelf" method.

### Model selection

Linear models. The linear regression model was estimated using the *lm* function of the *stats* package (R Core Team, 2019), with no additional hyper parameters. As multilevel models are not part of the *caret* package, they were implemented into *caret* using the *lmer* function from the *lme4* package version 1.1.21 (Bates et al., 2015), with no additional hyper parameters. The function call was different for *lmer*, as (1|SchoolID) was added to the equation in order to specify “school” as the grouping factor of the multilevel model. Linear support vector machines were estimated using the method *svmLinear* from the *kernlab* package (Karatzoglou et al., 2004), with grid search for the hyperparameter cost of constraint violation set at 0.001, 0.01, 0.1, 0.5, 0.9, and 1. Linear gradient boosting was estimated using the *xgboost* package version 0.90.0.2 (Chen et al., 2019), with the following hyperparameters: Number of boosting iterations: 25, 50, and 100; L1 and L2 regularization: 0, 0.01, 0.01, 0.1, and 1; learning rate: 0.05, 0.1, 0.3, and 0.6.

Non-linear models. The third degree polynomial regression was estimated with the same function as its linear counterpart, *lm* of the *stats* package (R Core Team, 2019) with no additional cross-validation parameters; the only difference to the linear regression was the model definition. More specifically, in the function call, the third degree was added to all continuous variables (i.e., the achievement variables and SES), but not to the categorical variables (i.e., migration status, language(s) spoken at home, and sex), with no interaction terms. Random forest models were estimated using the *ranger* package version 0.12.1 (Wright et al., 2020; the same package as used in Schiltz et al., 2018), with the following hyperparameters: number of randomly selected predictors of 2, 5, and 8; splitting rule of variance, extra trees, and maxstat; minimum node size of 5, 8, and 10. Neural networks were estimated using the *nnet* package (Venables & Ripley, 2002), with the following hyperparameters: number of hidden units: 1, 3, 5, and 10; weight decay: 0, 0.001, 0.1, 0.5, and 0.9. Third degree polynomial support vector machines were estimated using the method *svmPoly* from the *kernlab* package (Karatzoglou et al., 2004), with the following hyperparameters: Polynomial degree: 1, 2, and 3; distance measure for kernel: 0.001, 0.010, and 0.100; cost of constraint violation: 0.001, 0.01, 0.1, and 1. Similarly, radial support vector machines were estimated using the method *svmRadial* from the *kernlab* package (Karatzoglou et al., 2004), with the following hyperparameters: distance measure for kernel: 0.01, 0.05, 0.1, 0.5, and 1; cost of constraint violation: 0.001, 0.01, 0.1, 0.5, 0.9, and 1.
